# Photoprotection and Antiaging Activity of Extracts from Honeybush (*Cyclopia* sp.)—In Vitro Wound Healing and Inhibition of the Skin Extracellular Matrix Enzymes: Tyrosinase, Collagenase, Elastase and Hyaluronidase

**DOI:** 10.3390/pharmaceutics15051542

**Published:** 2023-05-19

**Authors:** Anna Hering, Justyna Stefanowicz-Hajduk, Magdalena Gucwa, Bartosz Wielgomas, Jadwiga Renata Ochocka

**Affiliations:** 1 Department of Biology and Pharmaceutical Botany, Medical University of Gdansk, 80-416 Gdansk, Poland; justynastef@gumed.edu.pl (J.S.-H.); magdalena.gucwa@gumed.edu.pl (M.G.); renata@gumed.edu.pl (J.R.O.); 2 Department of Toxicology, Medical University of Gdansk, 80-416 Gdansk, Poland; bartosz.wielgomas@gumed.edu.pl

**Keywords:** *Cyclopia* sp., skin, wound healing, mangiferin, hesperidin, tyrosinase, elastase, hyaluronidase, collagenase, antioxidant, anti-inflammation, plant extract, SPF, skin whitening effect

## Abstract

*Cyclopia* sp. (honeybush) is an African shrub known as a rich source of polyphenols. The biological effects of fermented honeybush extracts were investigated. The influence of honeybush extracts on extracellular matrix (ECM) enzymes responsible for the skin malfunction and aging process—collagenase, elastase, tyrosinase and hyaluronidase—was analysed. The research also included assessment of the in vitro photoprotection efficiency of honeybush extracts and their contribution to the wound healing process. Antioxidant properties of the prepared extracts were evaluated, and quantification of the main compounds in the extracts was achieved. The research showed that the analysed extracts had a significant ability to inhibit collagenase, tyrosinase and hyaluronidase and a weak influence on elastase activity. Tyrosinase was inhibited effectively by honeybush acetone (IC_50_ 26.18 ± 1.45 µg/mL), ethanol (IC_50_ 45.99 ± 0.76 µg/mL) and water (IC_50_ 67.42 ± 1.75 µg/mL) extracts. Significant hyaluronidase inhibition was observed for ethanol, acetone and water extracts (IC_50_ were 10.99 ± 1.56, 13.21 ± 0.39 and 14.62 ± 0.21µg/mL, respectively). Collagenase activity was inhibited effectively by honeybush acetone extract (IC_50_ 42.5 ± 1.05 μg/mL). The wound healing properties of the honeybush extracts, estimated in vitro in human keratinocytes (HaCaTs), were indicated for water and ethanol extracts. In vitro sun protection factor (SPF in vitro) showed medium photoprotection potential for all the honeybush extracts. The quantity of polyphenolic compounds was estimated with the use of high-performance liquid chromatography equipped with diode-array detection (HPLC-DAD), indicating the highest mangiferin contents in ethanol, acetone and *n*-butanol extracts, while in the water extract hesperidin was the dominant compound. The antioxidant properties of the honeybush extracts were estimated with FRAP (2,4,6-Tris(2-pyridyl)-s-triazine) and DPPH (2,2-diphenyl-1-picrylhydrazyl) tests, indicating their strong antioxidant activity, similar to ascorbic acid for the acetone extract in both tests. The wound healing abilities, estimation of SPF in vitro and the direct influence on selected enzymes (elastase, tyrosinase, collagenase and hyaluronidase) of the tested honeybush extracts were analysed for the first time, indicating a high potential of these well-known herbal tea for antiaging, anti-inflammation, regeneration and protection of the skin.

## 1. Introduction

Natural antioxidants are nowadays in demand due to their pro-health properties and abilities to protect against many serious systemic diseases. Nutritional deficiencies and life under constant stress cause the progressive destruction of the human body, which is exacerbated by the oxidative stress generated by many factors in the surrounding environment. Smog, UV radiation, smoking and consuming highly processed food lead to an increasing number of civilisation diseases [1]. Currently, there is a growing need for new and rediscovery of long-known plant sources of nutrients as protectors against oxidative stress. The search concerns not only nutraceuticals, but also sources of cosmetic ingredients that can protect the body against external free radicals and UV radiation [2,3,4].

*Cyclopia* spp. (Eng. honeybush, Fabaceae) can be utilised as a source with a unique composition of compounds with antioxidant properties: the plant may be used as an unprocessed plant material—green honeybush being utilised by the nutraceutical industry to produce supplements and antioxidant food additives—and as a fermented plant material to prepare tea beverages, popular not only because of the honey taste but also because of the antiaging properties. Extracts of both processed and unprocessed honeybush are utilised in the cosmetic industry, their applicability being confirmed by research, although fermented honeybush is more widely known and better distributed around the world [5,6,7,8].

Honeybush is one of the endemic species occurring only within in a part of the Cape Province in the Republic of South Africa. Honeybush can be obtained from wild areas or from plantations. The specific climatic conditions, including the high salinity of the soil, forced the plants to adapt their morphological and chemical compositions to survive. Utilised leaves and branches of honeybush are known as sources with high contents of polyphenolic compounds, which determine the biological activity of honeybush extracts. Many studies have focused mainly on the antioxidant, anticancer or phytoestrogen properties [9,10,11]. However, the honeybush also has numerous extremely important protective and regenerative properties that can be successfully utilised in cosmetology and dermatology [8,10,12]. Honeybush polyphenolic compounds work as a shield against skin degradation caused by UV radiation [7,13,14]. In addition, the plant is effective in the inhibition of wrinkle formation and the skin aging process [5,15]. Mangiferin, isomangiferin, hesperidin and vicenin-2—the main honeybush constituents—are highly pharmacologically active polyphenols, known also for their antioxidant and protective effects on cells [16,17,18,19].

Polyphenolic contents in honeybush extracts differ depending on the season, the plant material used (fermented or nonfermented) as well as the kind of extraction solution employed [20]. Despite the high polyphenolic contents in extracts, low intestinal absorption is a limiting factor for oral nutraceutical administration; therefore, other routes of administration should be considered [21,22,23]. In our previous study, we demonstrated that mangiferin and hesperidin are capable of passing through the human stratum corneum and permeating the epidermis and dermis, both from solutions and honeybush extracts. The ex vivo experiments indicated that hesperidin penetrates the skin from water and ethanol solutions with higher efficiency than from honeybush extracts. In addition, hesperidin indicates a lack of ability to interact with skin macromolecules, but, being unbound, may be capable of protecting cells against UV radiation and oxidative stress [24,25]. Mangiferin, in contrast to hesperidin, from honeybush extracts has shown a greater ability to penetrate the skin [21,24] and exhibited an ability to interact with skin extracellular matrix (ECM) molecules, incompetently inhibiting elastase and collagenase activity [21]. ECM compounds, such as collagen and elastin, are the structural and elasticity fibres of many organs and tissues [26,27], and they are also presented in abundant amounts among skin layers, where, with hyaluronic acid, they determine the skin functions [28,29,30]. The skin, which is the most external organ of the body, is not only the main target for UV radiation and extremal aging, but also exhibits internal diseases [31]. Besides the skin changes caused by ECM destruction, additional signs of skin aging are connected with the appearance of discoloration. Increase in melanin production as a result of the malfunctioning of melanocytes is mainly caused by the increased activity of tyrosinase enzymes. Moreover, highly reactive quinones decrease the functionality of skin macromolecules [30,32]. Dysfunction of ECM caused by oxidative stress and overproduction of enzymes responsible for macromolecule degradation leads to a decrease in the reconstruction and regeneration abilities of the skin. Mangiferin exhibited the best ability, among analysed honeybush extract constituents, to limit skin dysfunction and wrinkle formation [21,33,34,35], especially with the presence of other antioxidants from honeybush extracts [20].

Wound healing as an important marker of skin condition is a complex process, in which many abnormalities can be induced by the presence of free radicals [36,37]. Mangiferin and other polyphenols from honeybush that are strong antioxidants have a huge potential to be helpful during wound regeneration [38,39]. In addition, polyphenols are capable of absorbing both UV-A and UV-B radiation, which are responsible for DNA and skin macromolecule degradation [40,41]. Sunscreens sourced from nature, especially from the group of polyphenols, are in demand. Some plant extracts and antioxidant compounds, such as curcumin and quercetin, have anti-inflammatory activity via hyaluronidase inhibition and may limit the inflammation process around sunburnt or wounded skin [39,42].

The aim of this study was to demonstrate the antiaging properties of different honeybush extracts by estimation of their inhibition effects on the enzymes responsible for the degradation of ECM macromolecules: collagenase, elastase, hyaluronidase and tyrosinase. Although studies of the influence of honeybush extracts on the contents of elastin, collagen and hyaluronic acid in the skin have been conducted [5,6,7,13], no one has analysed the direct impact and differences in the inhibitory properties of water, ethanol, acetone and *n*-butanol extracts from fermented honeybush on the ECM enzymes. To quantify the polyphenolic contents of the main polyphenolic compounds—mangiferin, isomangiferin, hesperidin and vicenin-2—HPLC-DAD analysis was conducted. The antioxidant properties of honeybush extracts were assessed with the use of two tests: FRAP and DPPH tests. The skin protection and regeneration properties of the honeybush extracts were also evaluated in this work.

## 2. Materials and Methods

### 2.1. Materials

Collagenase from *Clostridium histolyticum*, neutrophil elastase, tricine buffer, N-[3-(2-Furyl) acryloyl]-Leu-Gly-Pro-Ala (FALGPA), N-Succinyl-Ala-Ala-Ala-p-nitroanilide (SANA), kojic acid, mangiferin, isomangiferin, hesperidin, vicenin-2, oleanolic acid, hyaluronidase (hyaluronidase from bovine testes, 400–1000 U/mg), tyrosinase (tyrosinase from mushroom), 3-(3,4-Dihydroxyphenyl)-L-alanine (L-DOPA), phosphate buffer (0.175 mM, pH 6.8), hyaluronic acid, bovine serum albumin (BSA), DPPH (2,2-diphenyl-1-picrylhydrazyl), TPTZ (2,4,6-Tris(2-pyridyl)-s-triazine), DMSO (dimethyl sulfoxide), ascorbic acid and phosphoric acid were sourced from the Sigma Chemical Co. (St. Louis, MO, USA), and TRIS-HCl, HCl (77 mM), NaCl, CaCl_2_, acetate buffer (acetic acid 79 mM, sodium acetate 24 mM, pH 3.75)—for enzymatic assays—and acetate buffer (0.3 M, pH 3.6)—for FRAP assays—were sourced from Avantor Performance Materials Poland S. A. FeCl_3_ × 6 H_2_O and HPLC-grade methanol and ethanol were purchased from P.O.Ch. (Gliwice, Poland).

### 2.2. Plant Material and Extract Preparation

The dried, fermented leaves and branches of *Cyclopia* sp. were purchased from KAWON-HURT Co., Gostyn, Poland. The plant material was used for extract preparation. Briefly: 15 g of plant material for each extract preparation was mechanically homogenised and extracted with 100 mL of the solvent (water, ethanol (50%, *v*/*v*), acetone (50% *v*/*v*) and *n*-butanol) with the use of an ultrasonic water bath (3× (50 Hz for 30 min)). The obtained extracts were evaporated under vacuum at 40 °C. The dry residues were lyophilised and stored before analysis at −20 °C. A representative sample of the plant material was deposited at the Department of Biology and Pharmaceutical Botany, Medical University of Gdansk, Poland.

The solvents used for the A–D extract preparations are listed in Table 1. The solvents water and ethanol 50% (*v*/*v*) were used, this being the easiest way to obtain homemade plant extracts used in everyday preparations. Acetone/water and *n*-butanol solvents were used to obtain extracts with high polyphenolic contents [43,44].

### 2.3. Chromatography

The HPLC system and the course and conditions of the analysis were described previously [21,24,45]. Briefly, the analyses were performed on a Dionex HPLC system (a Dionex P580 gradient pump, a Dionex TCC-100 column oven, a Dionex ASI-100T autosampler and a Dionex UltiMate 3000 Photodiode Array Detector (Dionex Corporation, Sunnyvale, CA, USA)). An ACE Excel C18-PFP (75 mm × 4.6 mm, 3 µm) column was used for separation and operated at 50 °C.

Multi-step gradient elution was applied to separate sample components using water (A) and acetonitrile (B) as a mobile phase, both containing 0.1% of HCOOH. The concentration of B was initially kept at 10% for 1 min, followed by a linear ramp to 20% over 29 min, then kept constant for 4 min and returned over 1 min to the initial conditions and left for 5 min before the next chromatographic run. The total analysis time was 40 min.

For the photodiode array detector, 225, 254, 280 and 360 nm wavelengths were utilised [46]. Prior to analysis, the samples were brought to room temperature, vigorously vortexed for 1 min and transferred into amber glass chromatography vials. Analytical standard preparation: individual stock solutions (1 mg/mL) of the analysed substances were prepared in DMSO or methanol depending on the solubility and were further stored in amber vials at +4 °C. Calibration standard solutions containing all the studied analytes were prepared in 20% methanol in water.

Identification of polyphenolics was achieved by comparing retention times and UV spectra with those of authentic standards.

Major validation parameters: limit of quantification (LOQ), linear range, accuracy and precision were established for the polyphenolic compounds that were present in the studied extracts: mangiferin, isomangiferin, vicenin-2 and hesperidin. Respective LOQs were within the range of 0.050–0.25 μg/mL, and calibration curves were linear (r > 0.99) in the range from LOQ to 100 μg/mL. Accuracy, expressed as an agreement between nominal and measured concentration, was 91–106%, depending on the analyte. Within- and between-day precision was in the range of 4.6–12.2%.

### 2.4. Tyrosinase Assay

A spectrophotometric tyrosinase assay was performed according to the method of Yagi et al. [47], with some modifications. L-DOPA was used as the substrate. The reaction mixture was composed of a phosphate buffer (0.175 mM, pH 6.8), 20 μL of tyrosinase (120 U) and different concentrations of extracts A–D (0–250 μg/mL). Reaction mixtures were pre-incubated with the extracts for 15 min at room temperature in 96-well plates (Biocom Systems, USA). The addition of L-DOPA started the reaction. Product formation changes were recorded spectrophotometrically at λ = 475 nm every 20 s for 20 min (Epoch BioTek System, USA). The control was composed of the phosphate buffer (0.175 mM, pH 6.8), an appropriate concentration of the extract and L-DOPA. Kojic acid was used as a standard. The results were analysed in the GraFit v.7.0 program (Erithacus Software) and Microsoft Excel.

### 2.5. Elastase Assay

A spectrophotometric elastase analysis was performed according to the method of Thring et al. [48], modified by Ochocka et al. [21]. The reaction mixture was composed of Tris-HCl buffer (pH 8.0), 1.0 μg/mL porcine pancreatic elastase and different concentrations of honeybush extracts A–D (0–800 μg/mL). Before addition of the substrate—SANA—the reaction mixtures were pre-incubated with the extracts for 15 min at room temperature in 96-well plates ((Epoch, BioTek System, Santa Clara, CA, USA). The addition of SANA started the reaction. Product formation changes with respect to p-nitroaniline were recorded spectrophotometrically at λ = 410 nm every 20 s for 20 min (Epoch, BioTek Instruments, Santa Clara, CA, USA). The results were analysed in the GraFit v.7.0 program (Erithacus Software, Wilmington House, High Street, East Grinstead, West Sussex, RH19 3AU, UK ) and Microsoft Excel. The control was composed of Tris-HCl buffer (pH 8.0), an appropriate concentration of the extract and SANA. Oleanolic acid was used as a standard.

### 2.6. Collagenase Assay

A spectrophotometric collagenase assay was performed according to the method of Thring et al. [48], modified by Ochocka et al. [21], with N-[3-(2-Furyl) acryloyl]-Leu-Gly-Pro-Ala (FALGPA) as the substrate. The reaction mixture was composed of 50 mM tricine buffer (pH 7.5 with 400 mM NaCl and 10 mM CaCl_2_), 0.8 mM FALGPA, 0.1 units of collagenase and different concentrations of extracts A–D (0–250 μg/mL). Reaction mixtures were pre-incubated with the extracts for 15 min at room temperature in 96-well plates (Biocom Systems). The addition of FALGPA started the reaction. Product formation changes were recorded spectrophotometrically at λ = 335 nm every 20 s for 15 min (Epoch BioTek System). The control was composed of 50 mM tricine buffer (pH 7.5 with 400 mM NaCl and 10 mM CaCl_2_), 0.8 mM FALGPA and an appropriate concentration of the extract. Oleanolic acid was used as a standard. The results were analysed in the GraFit v.7.0 program (Erithacus Software) and Microsoft Excel.

### 2.7. Hyaluronidase Assay

A colorimetric hyaluronidase assay was performed according to the method of Kaessler et al. [49], with some modifications. The analysis consisted of a ten-minute incubation of the sample with hyaluronidase (100 U/mL) dissolved in phosphate buffer (20 mM, pH 7.0), with the addition of NaCl (77 mM) and BSA (0.01% *v*/*v*) in a water bath at 37 °C. Addition of the substrate—hyaluronic acid dissolved in phosphate buffer (300 mM, pH 5.35)—started the reaction. Undigested hyaluronic acid was precipitated during the ten-minute incubation with bovine acid albumin (BSA 0.1% in sodium acetate 24 mM and acetic acid 79 mM, pH 3.75). Then, the spectroscopic measurement was performed at λ = 600 nm. The control was composed of the phosphate buffer (20 mM, pH 7.0), with the addition of NaCl (77 mM) and BSA (0.01% *v*/*v*), hyaluronic acid dissolved in phosphate buffer (300 mM, pH 5.35) and an appropriate concentration of the extract. Oleanolic acid was used as a standard. The obtained results were converted to the enzyme activity according to the equation:

Hyaluronidase activity = 100% − [ΔA Sample/ΔA Positive Control]

where “ΔA of the sample” refers to the reaction mixture with hyaluronidase, hyaluronic acid and a sample of the extract and “ΔA of the Positive Control” refers to the reaction mixture with hyaluronidase and hyaluronic acid, and the results were analysed in GraFit v.7.0 (Erithacus Software, Wilmington House, High Street, East Grinstead, West Sussex, RH19 3AU, UK) and Microsoft Excel.

### 2.8. DPPH Assay

The spectrophotometric DPPH radical scavenging assay was performed [50]. Briefly: 100 µL of different concentrations of the extracts, dissolved in water, were mixed with 100 µL of 0.06 mM DPPH methanolic solution and incubated at room temperature in the dark for 30 min. The absorbance was analysed spectrophotometrically at λ = 510 nm using a 96-well microplate reader (Epoch, BioTek System, Winooski, VT, USA). The control was composed of DPPH and water. Ascorbic acid was used as the standard. DPPH inhibition was calculated according to the following equation:

DPPH Inhibition (%) = [(A_control_ − A_extract_)/A_control_] × 100%

The antioxidant activity of the extracts was shown as the IC_50_ value (the concentration of the analysed extract or the standard substance that caused a decrease in the non-reduced form of the DPPH radical by 50%). The IC_50_ value was calculated with GraFit v.7.0 (Erithacus Software).

### 2.9. FRAP Assay

The reducing ability of the analysed honeybush extracts was determined with the FRAP test, based on the reduction of Fe^+3^ to Fe^+2^ [51]. The course of the analysis: 30 μL of serial dilutions of the extracts and the standard substance (ascorbic acid), placed in a 96-well plate, were mixed with 170 μL of the freshly prepared reaction mixture (0.3 M acetate buffer: 10 mM TPTZ in 40 mM HCl:20 mM FeCl_3_ × 6 H_2_O in a ratio of 10:1:1). The plate was incubated at room temperature for 20 min, after which the absorbance was read at 593 nm. The percentage of reduced iron ions was read from the calibration curve plotted for the standard ascorbic acid (1–1000 µg/mL). Then, the IC_50_ value—the concentration of the analysed extracts that reduced iron ions by 50%—was calculated with GraFit v.7.0 (Erithacus Software). The control was composed of the reaction mixture and water.

### 2.10. Estimation of SPF

Methanol aliquots of the extracts were prepared in the concentration range of 62.5–1000 µg/mL and analysed spectrophotometrically (Epoch, BioTek System, Winooski, VT, USA). The solutions were placed in a quartz cuvette (1 cm length), and the absorbance spectra were recorded in triplicate (λ = 280–500 nm, with 5 nm intervals). Methanol was used as a blank. Sun photoprotection factor in vitro was estimated according to the Sayre method, modified by Mansur [52,53].

SPF in vitro was calculated according to the following equation:

SPF in vitro *=* CF × ∑ ^320^ _290_ EE(λ) × I(λ) × A(λ)

where “EE(λ)” refers to the erythemal effect spectrum at wavelength λ; “I(λ)” refers to the solar intensity spectrum at wavelength λ and the values of EE (λ) × I were constant; “A(λ)” refers to the absorbance of the extract solution determined by UV spectrophotometry at a wavelength (λ); and “CF” refers to the correction factor (CF = 10).

### 2.11. Wound Healing Assay

The human keratinocyte (HaCaT) cells (obtained from the American Type Culture Collection (ATCC), USA) were seeded in 12-well plates (1 × 10^5^ cells/well) and incubated for 24 h in a humidified 5% CO_2_ incubator (37 °C) in Dulbecco’s Modified Eagle’s Medium (DMEM) supplemented with 10% (*v*/*v*) fetal bovine serum (FBS), 100 units/mL of penicillin and 100 µg/mL of streptomycin (Merck Millipore, Burlington, MA, USA). Then, a scratch at the bottom of each well was made with tips, and different *Cyclopia* sp. extracts were added to the cells and incubated for up to 96 h. After treatment, the cells were washed and analysed under a microscope (magnification × 100) (Leica, Switzerland). All samples were compared to the untreated control cells.

### 2.12. Statistical Analysis

Statistical data were analysed using the STATISTICA 12.0 software package (StatSoft Inc., Tulsa, OK, USA). All data are expressed as mean values ± standard deviations (SDs). For comparison studies, one-way ANOVA with post hoc Tukey’s tests were performed. Statistical significance was set at *p* < 0.05.

### 2.13. Sample Availability

Samples of the plant material and compounds are available from the authors.

## 3. Results

### 3.1. HPLC

**Figure 1:**
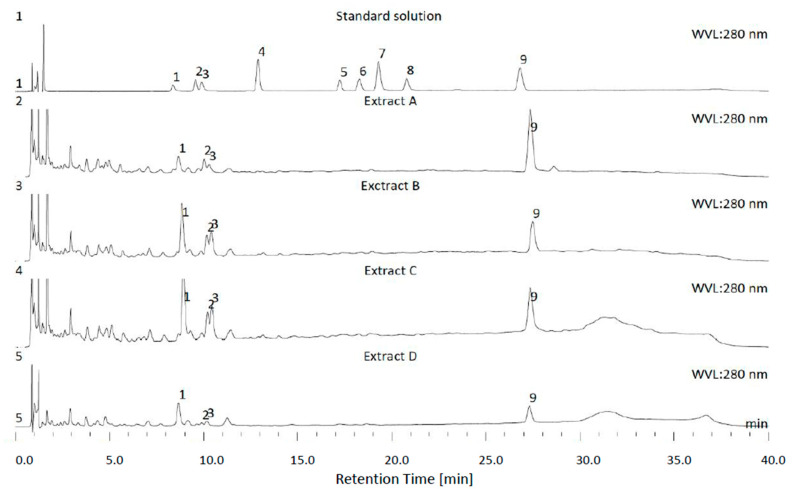
HPLC-UV chromatograms of standards (standard solution, 10 μg/mL) and water (extract A), 50% ethanol/water (*v*/*v*) (extract B), 50% acetone/water (*v*/*v*) (extract C) and *n*-butanol (extract D) honeybush (*Cyclopia* sp.) extracts. Detection wavelength (WVL): 280 nm. Peaks annotations: 1—Mangiferin; 2—Isomangiferin; 3—Vicenin-2; 4—Vicenin-1; 5—Vicenin-3; 6—Eriocitrin; 7—Rutin; 8—Scolimoside; 9—Hesperidin.

The HPLC-DAD method was utilised to quantitatively analyse the four main polyphenols from honeybush extracts known for their biological activity: mangiferin, isomangiferin, hesperidin and vicenin-2 (Figure 1) [10,11,20,46].

**Figure 2:**
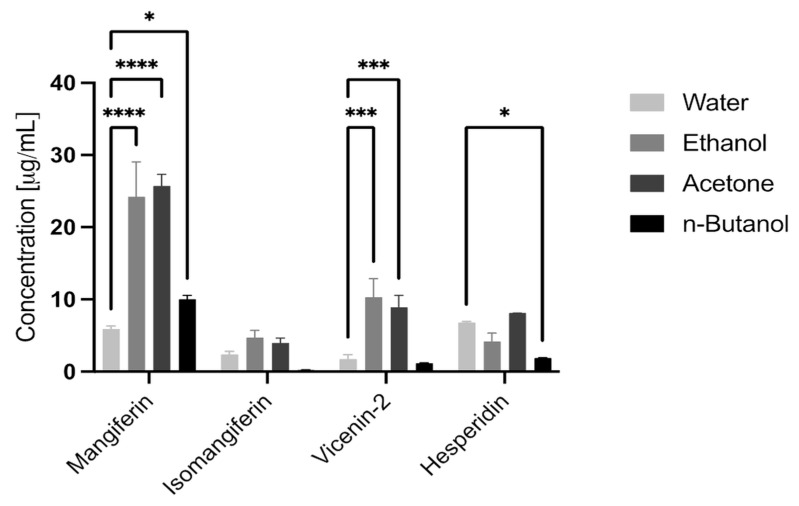
The contents of mangiferin, isomangiferin, hesperidin and vicenin-2 in the honeybush extracts. Two-way ANOVA with Dunnett comparison for multiple comparisons. Water extract sets were used as a reference. Only differences with *p* < 0.05 are shown on the graph (* *p* < 0.05, *** *p* < 0.001 and **** *p* < 0.0001).

The results (Table 2, Figure 1 and Figure 2) indicated that in extracts A-D all analysed polyphenols were detected. The highest concentrations were present in extracts B and C, while extract A followed by extract D had lower contents. Mangiferin exhibited predominating amounts, especially in extracts B and C. In these, both extracts vicenin-2 and isomangiferin were also present at the highest concentrations. The most significant amounts of hesperidin were observed in extracts A and C. Surprisingly, the water extract (A) exhibited higher concentrations of isomangiferin and vicenin–2 than *n*-butanol (extract D).

### 3.2. DPPH (2,2-Diphenyl-1-Picrylhydrazyl) Scavenging Analysis and FRAP (2,4,6-Tris(2-Pyridyl)-s-Triazine) Assay

The DPPH and FRAP tests are widely used to assess the antiradical ability and ion Fe^3+^ reduction of raw natural materials. Although honeybush plant material has been analysed with different antioxidative tests, including DPPH and FRAP [8,10], we conducted these assays to establish the antioxidant capacity and thus judge the quality of the plant material and the resulting extracts. The reason for the different biological activities of plant extracts is the fact that polyphenols are highly unstable and sensitive to weather and environmental conditions during plant growth and development, as well as transport and storage time [54].

The data presented in Table 3 indicate the high ability of the analysed plant material from honeybush to reduce both DPPH radicals and Fe^3+^ ions. The high antioxidant potency of the *Cyclopia* sp. extracts corresponded to their contents of polyphenolic compounds (Table 2). Generally, the higher the polyphenol concentration in the extract, the better the results obtained in both tests. The highest activity was observed for extract C (acetone), the IC_50_ value for which was similar to the IC_50_ of the standard ascorbic acid (Table 3).

The lowest ability to reduce Fe^3+^ ions and to scavenge DPPH radicals was indicated for extract D (*n*-butanol), which has the lowest analysed polyphenol concentration.

Extracts A and B presented similar antioxidant properties in both tests, with the slight predominance of the ethanol extract (B). Thus, the results indicate that the antioxidant activity of the extracts from honeybush is caused not only by the presence of mangiferin but also by other compounds in the extracts.

### 3.3. The Influence of Honeybush Extracts on Enzyme Activity

Honeybush extracts were investigated in order to estimate their ability to inhibit the activity of enzymes: tyrosinase, elastase, collagenase and hyaluronidase. All the obtained IC_50_ values are summarised in Table 4.

#### 3.3.1. The Influence of Honeybush Extracts on Tyrosinase Activity

Honeybush extracts were investigated for their antityrosinase activity using L-DOPA as the substrate and kojic acid as a control.

Among the analysed honeybush extracts, A, B and C indicated an inhibitory effect on tyrosinase activity (Table 4). Water (extract A) and ethanol (extract B) indicated tyrosinase inhibition properties three and two times weaker (IC_50_ 67.42 ± 1.75 and 45.99 ± 0.76 µg/mL, respectively) than that of kojic acid (IC_50_ 21.99 ± 0.8 µg/mL). Only extract C (acetone extract) showed an activity similar to the standard inhibitory activity respecting tyrosinase (IC_50_ 26.18 ± 1.45 µg/mL). Despite the increase in concentration in the probes, extract D did not affect tyrosinase activity.

#### 3.3.2. The Influence of Honeybush Extracts on Elastase Activity

Honeybush extracts were investigated for their antielastase activity using SANA as the substrate and oleanolic acid as a control.

Extracts B, C and D exhibited weak inhibition properties regarding elastase activity (Table 4). None of the analysed extracts reached the IC_50_ of oleanolic acid (19.34 ± 0.78 µg/mL). The strongest inhibition was presented for *n*-butanol extract D, with an IC_50_ of 666.27 ± 6.51 µg/mL, and a weaker influence on elastase activity was indicated for acetone extract C (IC_50_ 750.06 ± 3.54 µg/mL) and extract B (IC_50_ 1104.97 ± 47.45 µg/mL). Despite the increasing concentration of the water extract A (until 1.5 mg/mL in a reaction mixture), elastase activity was not decreased in the analysed reaction conditions.

#### 3.3.3. The Influence of Honeybush Extracts on Collagenase Activity

Honeybush extracts were investigated for their anticollagenase activity using FALGPA as the substrate and oleanolic acid as a control.

All the tested extracts exhibited an ability to inhibit collagenase activity (Table 4), although their IC_50_ values were smaller in comparison to that of oleanolic acid (IC_50_ 25.66 ± 0.39 μg/mL). Extract D had the least effect on collagenase activity (IC_50_ 420.01 ± 2.77 μg/mL), while the IC_50_ of acetone extract (C) was only two times lower (IC_50_ 42.5 ± 1.05 μg/mL) than the standard substance. Water (A) and ethanol (B) extracts from honeybush exhibited similar inhibition of collagenase activity, with IC_50_ values of 77.25 ± 2.74 and 72.15 ± 0.24 μg/mL, respectively, which were about three times weaker than that of oleanolic acid.

#### 3.3.4. The Influence of Honeybush Extracts on Hyaluronidase Activity

Honeybush extracts were investigated for their antihyaluronidase activity using hyaluronic acid as the substrate and oleanolic acid as a control.

All the extracts of honeybush exhibited stronger inhibitory effects on the activity of hyaluronidase than the standard substance—oleanolic acid (IC_50_ 51.05 ± 0.53 μg/mL). The results of the calculated IC_50_ values for the extracts from honeybush listed in Table 4 indicated the strongest antihyaluronidase activity for extracts B (ethanol) > C (acetone) > A (water) (IC_50_ 10.99 ± 1.56, 13.21 ± 0.39 and 14.62 ± 0.21 µg/mL, respectively). Among the extracts, the weakest influence on hyaluronidase activity, although still stronger than the standard, was demonstrated for extract D, with IC_50_ 38.21 ± 0.79 µg/mL.

### 3.4. The Sun Protection Factor In Vitro of the Honeybush Extracts

For the analysed extracts, UV spectra (λ 275–500 nm) were determined for the series of dilutions (62.5–1000 µg/mL).

**Figure 3:**
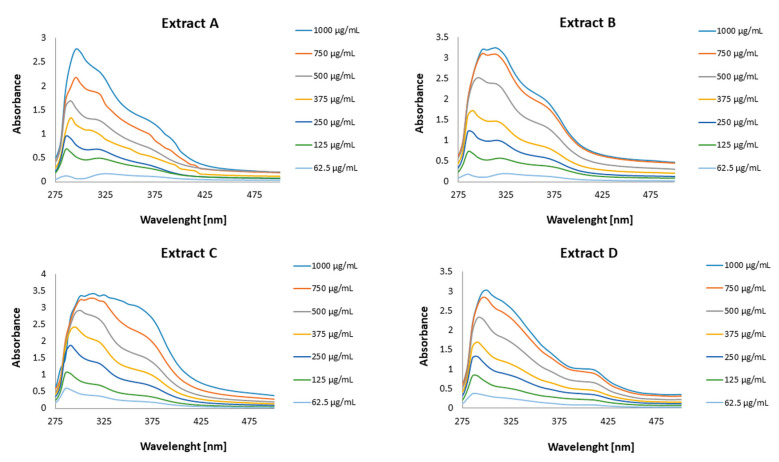
UV absorbance of the honeybush extracts: (**A**) water, (**B**) ethanol (50% *v*/*v*), (**C**) acetone (50% *v*/*v*) and (**D**) (*n*-butanol).

The UV spectra for the extracts from honeybush are presented in Figure 3. The results revealed wavelength-absorbance changes dependent on the concentration of the extracts. A high level of absorbance was confirmed for all the extracts, with the highest absorbance in the range of 280–380 nm, indicating potential protection against both UVA (320–400 nm) and UVB (280–320 nm). The highest absorbance in the examined wavelength range was shown by extract C, followed by extract B.

The sun photoprotection factors of the honeybush extracts are presented in Table 5 and classified according to European Commission recommendations:

- No protection SPF in vitro ≤ 5.9;
- Low protection 6.0 ≤ SPF in vitro ≤ 14.9;
- Medium protection 15.0 ≤ SPF in vitro ≤ 29.9;
- High protection 30.0 ≤ SPF in vitro ≤ 59.9 [55].

The honeybush extracts at concentrations lower than 122 µg/mL presented no SPF in vitro protection. With the increase in extract amounts, the SPFs in vitro increased as well. Low SPF in vitro was indicated for extracts A, B and D at a concentration of 375 µg/mL and for extract A at a concentration of 500 µg/mL. Among the analysed extracts, extract C presented medium SPF in vitro abilities at concentrations above 375 µg/mL, and at a concentration of 1000 µg/mL had close to a high SPF in vitro (27.81 ± 0.03). Similar, smaller SPF in vitro values were indicated for extracts B and D. The weakest protection was shown for extract A, with medium protection in the concentration range of 750–1000 µg/mL.

### 3.5. The Effect of Honeybush Extracts on Wound Healing In Vitro

**Figure 4:**
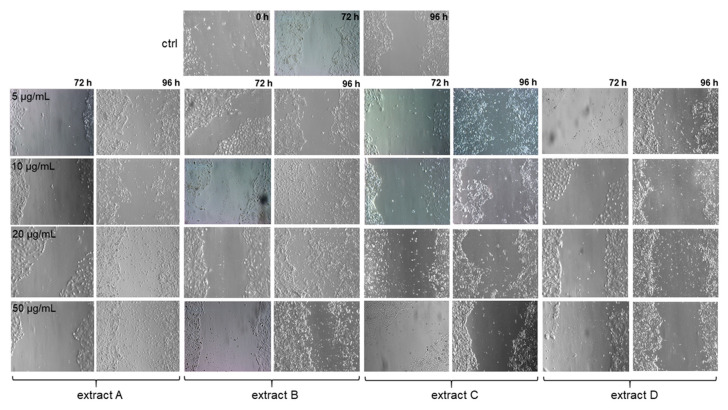
The effects of the honeybush extracts on migration of HaCaT cells and wound healing. The cells were incubated with the extracts (A, B, C and D) at concentrations of 5–50 µg/mL for up to 96 h and analysed under a microscope (magnification × 100).

To estimate the properties of the honeybush extracts on wound healing in vitro, we used HaCaT cells and treated them with different concentrations of the extracts for up to 96 h. The sizes of the spaces at the bottom of each well at the beginning of the experiment were observed under a microscope and compared with the untreated control cells after 72 and 96 h of incubation. We observed migration of the cells in the controls and the overgrowing of gaps (Figure 4). Regarding samples with the extracts, we observed higher migration of HaCaT cells in the wells with extracts A and B than in the wells with extracts C and D in the range of concentrations used. The stimulation effect of extracts A and B on wound healing was well visible after 96 h of incubation in comparison to the control. Our preliminary results indicate that these extracts (A and B) may have potential to heal wounds; however, further molecular and especially in vivo studies should be conducted.

## 4. Discussion

In our study, we estimated the effects of different *Cyclopia* sp. extracts on the activity of ECM enzymes, the wound healing process and SPF in vitro. The experiments were preceded by quantification of the main secondary metabolites dominant in honeybush with proven biological activities. The quality of the prepared extracts was analysed with respect to antioxidant power, with the use of two tests: DPPH and FRAP tests.

The results indicated that the predominant compound in the analysed extracts from honeybush was mangiferin, followed by hesperidin and vicenin-2. Isomangiferin was also detected in all the analysed extracts, though in smaller amounts. The highest quantities of the analysed polyphenols were detected in acetone and ethanol extracts, while the lowest was detected in *n*-butanol extract. DPPH and FRAP assays confirmed that the acetone and ethanol extracts were the most capable of fighting DPPH radicals and inhibiting ferric reduction. Water and *n*-butanol extracts presented weaker antioxidant properties. These results are in agreement with the amounts of polyphenols in the extracts. Our research confirmed the good quality of the tested plant material.

Next, in the experiments on the ECM enzymes, all of the analysed enzymes—collagenase, elastase, hyaluronidase and tyrosinase—were inhibited by the honeybush extracts.

In the case of the first tested enzyme—tyrosinase—the acetone honeybush extract could inhibit its activity with an IC_50_ similar to that of kojic acid, which is known as a strong tyrosinase inhibitor [56]. A weaker inhibition was detected for the ethanol and water extracts. High amounts of polyphenols, including mangiferin, the main compound in honeybush extract and a strong antioxidant, was detected in the most antityrosinase-active extracts. The exception was the n-butanol extract, which, despite the higher content of mangiferin compared to the aqueous extract, showed no tyrosinase activity. Probably, in this extract, apart from mangiferin, other compounds were extracted which have affinity for tyrosinase in a place other than the active site and block mangiferin-tyrosinase interaction [8,10,56]. Thus, extract D (*n*-butanol) should be examined in more detail in terms of chemical composition. Honeybush extracts are known as a good source of antioxidants [10], which was also confirmed in our analysis. The unique antioxidant compositions in honeybush extracts can protect the skin macromolecules and fight reactive quinones generated in L-DOPA degradation [32]. This suggests that the tested extracts in living organisms may influence tyrosinase activity even more effectively than kojic acid (including *n*-butanol extract) and that they can be utilised to limit skin discoloration problems.

Despite the fact that mangiferin is capable of entirely inhibiting elastase [21], honeybush extracts can cause a minor elastase activity reduction. The influence of the honeybush extracts on elastase activity is probably not only connected with mangiferin; other compounds present in the extracts may reduce the influence of mangiferin on the enzyme. Surprisingly, the *n*-butanol extract turned out to be the most reactive against elastase, while towards the other enzymes tested, it showed the weakest inhibition abilities. Further studies of the qualitative composition of the *n*-butanol extract could clarify this hypothesis.

Our previous collagenase inhibition analysis indicates that mangiferin is capable of entirely inhibiting collagenase [21]. Although the mangiferin contents in ethanol and acetone extracts were similar, the collagenase inhibition properties of the acetone extract were almost twice as strong as those of the ethanol and water extracts.

The analysed extracts exhibited a very significant inhibitory effect on the activity of hyaluronidase, the enzyme responsible for the degradation of hyaluronic acid, and in consequence lower skin hydration and induction of the inflammation process [30,47]. The strongest influence on hyaluronidase activity was demonstrated by the ethanol extract, with only small, weaker effects for the acetone > water > *n*-butanol extracts. Their IC_50_ values were much lower than that of the positive control—oleanolic acid. Thus, all the analysed extracts may be useful in fighting hyaluronic acid degradation due to aging as well as skin inflammation conditions caused by the increased activity of hyaluronidases. It should also be noted that hyaluronidases show very high tissue specificity [57]; thus, the utility of honeybush extracts in fighting inflammation in other organs and tissues should be analysed under different reaction (pH) conditions or via in vivo studies. Our research demonstrated that the *n*-butanol extract from honeybush exhibited low amounts of the analysed polyphenolic compounds and presented poor antioxidant properties in comparison with the water, ethanol and acetone extracts. The *N*-butanol extract also exhibited no ability to influence tyrosinase activity. Although the extract had a lower ability to inhibit collagenase than the other extracts from honeybush, it was proved that it can inhibit hyaluronidase, reaching an IC_50_ lower than the standard substance.

Our results indicate that the influence of honeybush extracts on ECM enzyme activities is more connected to their antioxidant properties than to the amounts of single polyphenols, such as mangiferin. The mangiferin in honeybush extracts occurs with other secondary compounds which can act synergistically with or antagonistically to mangiferin and thus change the mangiferin-enzyme affinity. Polyphenols may interact with the enzyme by electron or hydrogen transfer as well as chelating transition metals. Despite the high amounts of the potential inhibitor, the extracts may contain substances with higher affinity to the enzyme and which interact with it in places other than the active site, causing a change in the conformation of the enzyme and preventing the potential inhibitor from accessing the active site [58,59].

The extracts from plants characterised by a high ability to scavenge free radicals and protect ECM enzymes are often starting points for further research. Such an example is an extract of *Thunbergia laurifolia* (Acanthaceae), which exhibits both significant antioxidant properties and inhibits hyaluronidase and collagenase [60]. *Salicornia europaea* (Amaranthaceae) extract inhibits elastase and hyaluronidase, while *Spartina anglica* (Poaceae) could be successfully used in cosmetics to inhibit the activity of tyrosinase [61].

There are not many plant extracts with unique chemical compositions sufficient to inhibit the enzymes responsible for skin decomposition (collagenase and elastase), overpigmentation (tyrosinase), hydration and inflammation (hyaluronidase). Such an example is the ethanol extract from the leaves of *Euphorbia characias*, which presents biological effects on the skin similar to those of honeybush extracts. Despite the low skin absorption of this extract, the nanoparticles were utilised to enhance transdermal delivery [62,63].

The effects of honeybush extracts after oral and topical administration on the skin of mice showed antiwrinkle, anti-inflammatory and UV-protective effects (13,15), but the underlying mechanisms of the effects of honeybush extracts on ECM macromolecules have not been explained.

The tests performed previously by our team indicated that mangiferin and hesperidin from honeybush extracts may pass the stratum corneum and permeate the epidermis and dermis [21,24]. In addition, mangiferin has the ability to inhibit collagenase, elastase and tyrosinase [21,64].

The extracts from honeybush also exhibited in vitro wound healing properties that also require further and more detailed analysis. However, our research shows that water and ethanol extracts have high potential in supporting wound regeneration, especially considering that both extracts can be prepared and used at home. In addition, as our research has proved, all the extracts showed photoprotection abilities against both UVA and UVB solar radiation. The highest SPF in vitro was obtained for the acetone extract, with a slightly lower SPF in vitro being obtained for the ethanol extract.

Honeybush extracts and their single constituents are being intensively investigated, with promising results, for their UV protection and sunscreen utility [5,13,15,41]. Furthermore, their abilities to inhibit collagenase, hyaluronidase and tyrosinase, as well as improve wound healing, confirm the potential for the application of honeybush extracts in cosmetology and dermatology.

## 5. Conclusions

The above results of the analyses indicate that high polyphenolic contents in extracts from honeybush are responsible for their potential in the protection of macromolecules involved in skin structure, melanin distribution and hydration. More in-depth analyses should be focused on *n*-butanol, acetone and ethanol extracts regarding the skin regeneration and photoprotection properties of these extracts from honeybush.

## Figures and Tables

**Table 1:** The solvents used for the honeybush extract preparations.

Honeybush Extract	Solvents Used for the Extract Preparations
A	Water
B	50% Ethanol/water (*v*/*v*)
C	50% Acetone/water (*v*/*v*)
D	*n*-Butanol

**Table 2:** Concentrations of mangiferin, isomangiferin, vicenin-2 and hesperidin in extracts A–D (µg/mL).

	Water (A)		Ethanol (B)		Acetone (C)		*n*-Butanol (D)	
	Mean	SD	Mean	SD	Mean	SD	Mean	SD
Mangiferin	5.91	0.42	24.23	4.82	25.71	1.62	10.02	0.54
Isomangiferin	2.38	0.45	4.71	1.00	3.98	0.67	0.24	0.06
Vicenin-2	1.74	0.65	10.32	2.58	8.92	1.62	1.18	0.07
Hesperidin	6.80	0.16	4.19	1.15	8.10	0.03	1.87	0.07

**Table 3:** DPPH and FRAP scavenging assays with *Cyclopia* sp. extracts and vitamin C as standard.

Method	*Cyclopia* Extract	A	B	C	D	Standard Ascorbic Acid
DPPH	IC_50_ (µg/mL) *	28.83 ± 2.22 ^a^	18.87 ± 0.69 ^ab^	7.95 ± 0.56 ^b^	65.15 ± 1.22	7.11 ± 0.04
FRAP	IC_50_ (µg/mL) *	6.8 ± 0.41 ^cd^	5.5 ± 0.7 ^ce^	4.75 ± 0.24 ^de^	18.7 ± 0.15	4.19 ± 0.64

* The results are presented as mean values ± standard deviations (SDs) obtained in two independent analyses, three repetitions in each (*n* = 6). Significant differences among the results were indicated as “a, b, c, d, e” (one-way ANOVA with post hoc Tukey’s test, *p* < 0.05).

**Table 4:** The IC_50_ values of *Cyclopia* sp. extracts (extract A (water), B (ethanol 50%, *v*/*v*), C (acetone 50% *v*/*v*) and D (*n*-butanol)) on ECM enzymes.

IC_50_ (µg/mL)	Extract A	Extract B	Extract C	Extract D	Standard Oleanolic Acid *	Standard Kojic Acid *
Tyrosinase	67.42 ± 1.75	45.99 ± 0.76	26.18± 1.45	n.r	n.t.	21.99± 0.8
Elastase	n.r.	1104.97 ± 47.45	750.06 ± 3.54	666.27 ± 6.51	19.34 ± 0.78	n.t.
Collagenase	77.25 ± 2.74	72.15 ± 0.24	42.5 ± 1.05	420.01 ± 2.77	25.66 ±0.39	n.t.
Hyaluronidase	14.62 ± 0.21	10.99 ± 1.56	13.21 ± 0.39	38.21 ± 0.79	51.05± 0.53	n.t.

n.t.—not tested; n.r.—not reached. * These compounds were used as a standard; the results were obtained from three independent experiments with three repetitions (*n* = 9) and are presented as mean values with ± standard deviations (SDs). All the results were statistically significantly different in comparison to the control (oleanolic acid and/or kojic acid).

**Table 5:** The SPFs in vitro of *Cyclopia* sp. extracts.

Extract Concentration (µg/mL)	Extract A	Extract B	Extract C	Extract D
62.5	1.50 ± 0.03	1.89 ± 0.14	4.34 ± 0.04	3.35 ± 0.01
122	4.75 ± 0.05	5.35 ± 0.06	7.39 ± 0.01	5.98 ± 0.46
250	6.55 ± 0.03	9.09 ± 0.07	13.38 ± 0.41	9.33 ± 0.03
375	9.88 ± 0.05	13.12 ± 0.07	18.99 ± 0.04	12.19 ± 0.42
500	12.18 ± 0.08	20.72 ± 0.03	24.03 ± 0.28	17.64 ± 0.02
750	17.11 ± 0.19	25.75 ± 0.20	26.89 ± 0.01	22.84 ± 0.16
1000	21.97 ± 0.05	26.62 ± 0.09	27.81 ± 0.03	24.69 ± 0.04

## Data Availability

All data are included in the manuscript.
